# Consideration of health literacy in implementation science

**DOI:** 10.1017/cts.2021.775

**Published:** 2021-04-08

**Authors:** Hae-Ra Han, Deborah Wu, Janiece Taylor, Rebecca Wright, Martha Abshire

**Affiliations:** 1 Johns Hopkins School of Nursing, Baltimore, MD, USA; 2 Johns Hopkins Bloomberg School of Public Health, Baltimore, MD, USA

**Keywords:** Health literacy, evidence-based practice, implementation science, stakeholders

It has been widely stated that evidence-based practices (EBPs) take on average 17 years to be incorporated into routine clinical practice, with only about half of EBPs ever reaching widespread clinical adoption [1,2]. There is a growing interest in shortening the time lag between health research and translation into routine care for a public health impact. Implementation science is the scientific study of strategies to promote the uptake of research findings and other EBPs into real-world, general clinical practice with sustained public health benefits [3,4]. Broader than traditional clinical research in scope, implementation science requires involvement of diverse stakeholders who are not routinely part of clinical trials. In particular, health system stakeholders, including affected communities and operational partners such as administrators or health system managers and frontline health workers are key players in the conduct of implementation research projects [5]. Nevertheless, due to diverse educational backgrounds, clinical experiences, and expertise, bringing together these stakeholders often presents a critical challenge. Clear communication is an essential part of promoting a collaborative effort, and understanding health literacy can be a catalyst to address this challenge.

The Institute of Medicine report, Health Literacy: A Prescription to End Confusion, defines health literacy as “*the degree to which individuals have the capacity to obtain, process, and understand basic health information and services needed to make appropriate health decisions* [6].” More recent definitions focus on specific skills needed to navigate the health system and the importance of clear communication between health care providers and their patients [7]. The US Department of Education’s 2003 National Assessment of Adult Literacy – the most comprehensive assessment of adult literacy and the first-ever national assessment of health literacy [8] – revealed that only 12 percent of US adults are proficient enough in health literacy to understand and use printed health information effectively [9]; more than a third of US adults are in the “basic” or “below basic” health literacy groups, which means they may fail to understand most health information. Adding to these challenges, twenty-five million Americans (8.7 percent) have limited English proficiency [10]. There is a strong association between limited English proficiency and low health literacy [11,12]. Taken together, these statistics underscore the relevance of health literacy within implementation science, which requires clear communication to develop shared understanding among increasingly diversifying stakeholders as key players in the design and conduct of the research – patients, caregivers, frontline clinicians, administrators, and researchers.

We conducted a scoping review to better understand how published implementation science studies have addressed health literacy. A PubMed search was done to identify articles published from the inception through December 22, 2020 in English. We used broad search terms, “health literacy” and “implementation science” to identify potential articles in which both topics were addressed. The search resulted in a total of 18 articles; more than two-thirds (*n* = 14) were published in the last 5 years. The articles identified discussed a variety of interventions across different health conditions including HIV, cancer, mental illnesses, hypertension, diabetes, spinal cord injuries, and sickle cell disease. Health literacy was incorporated in one of three ways: (1) as a consideration when developing interventions, (2) as a factor in successful implementation of interventions, or (3) as an outcome the intervention sought to impact. The degree to which health literacy was incorporated, however, varied and was rarely a focal point.

We found three of the articles, which exemplified future directions for the integration of health literacy in implementation science. Specifically, Bohkhour et al. [13], Davis et al. [14], and Houston et al. [15] observed greater pre- and postintervention improvements in subgroups of participants with initially lower health literacy scores (measured at baseline using either Rapid Estimate of Adult Literacy in Medicine or Short Test of Functional Health Literacy). These three articles not only acknowledged the role of health literacy in successful intervention implementation but also sought to improve health literacy by incorporating it into the intervention design in a manner that tailors information appropriately to the population of interest. By stratifying outcome data according to health literacy scores, health literacy could be analyzed as a factor for successful implementation for achieving improved health outcomes.

Additional opportunities for addressing health literacy include understanding the role of health literacy in the implementation setting, where confusion and misunderstanding are likely to occur. One of the most popular implementation research frameworks, the Consolidated Framework for Implementation Research [16], offers multiple implementation contexts to consider for implementation of health interventions. One of them is the “inner setting” of organizations, which addresses characteristics and features of the implementing organization, and has been closely associated with implementation outcomes and the quality of care [17,18]. For example, how healthcare systems address varying levels of health literacy of their patients is called organizational health literacy [7]. There is a growing appreciation that health literacy is the byproduct of the demands that health systems or organizations place on individuals and the specific healthcare system where care is provided or health interventions are implemented. A health literate organization is an ideal setting to conduct an implementation research project as health literacy is a value and actively promoted mission within the organization [7]. Future implementation research should consider organizational health literacy as one of the key inner setting characteristics and at minimum, incorporate adequate assessment tools (e.g., Health literate healthcare organization 10-item questionnaire [19]) as part of the evaluation plan.

Healthcare environments around the world are increasingly dynamic, resource-constrained, and interconnected – and are driven by equally complex political and economic environments. Accordingly, maximizing healthcare value has become a policy imperative globally [20]. To this end, implementation science will become even more critical for promoting value-based health programs. As the focus on implementation science continues, health literacy can serve as an innovative and disruptive force that creates a new value equation for EBPs and implementation science.
